# Association analysis of *APO* gene polymorphisms with ischemic stroke risk: a case-control study in a Chinese Han population

**DOI:** 10.18632/oncotarget.15549

**Published:** 2017-02-20

**Authors:** Rongjun Xiao, Shuaiqi Sun, Jiayi Zhang, Yongri Ouyang, Ning Zhang, Min Yang, Tianbo Jin, Ying Xia

**Affiliations:** ^1^ Department of Neurosurgery, Affiliated Haikou Hospital of Xiangya Medical College of Central South University, Haikou People's Hospital, Haikou, Hainan, China; ^2^ School of Life Sciences, Northwest University, Xi'an, Shaanxi, China

**Keywords:** APO, ischemic stroke, gene polymorphisms, case-control study, Chinese Han population

## Abstract

This study aimed to assess the association of *APO* gene polymorphisms and ischemic stroke risk in a Chinese Han population. In this case-control study, we genotyped 14 single nucleotide polymorphisms (SNPs) in 3 *APO* genes in 488 cases and 503 controls using Sequenom Mass-ARRAY technology and evaluated their association with ischemic stroke using the χ2 and genetic model analysis. In the allelic model analysis, we determined three SNPs were significantly associated with ischemic stroke: rs693 with a *p* value of 0.042 (OR = 1.406; 95%CI = 1.011-1.956), rs651821 with a *p* value of 0.007 (OR = 0.760; 95%CI = 0.622-0.929) and rs662799 with a *p* value of 0.006 (OR = 0.755; 95%CI = 0.618-0.923). In the genetic model analysis, we found the minor allele “A” of rs693 was associated with an increased ischemic stroke risk in the additive model and dominant model. The minor allele “C” of rs651821 was associated with a decreased ischemic stroke risk in the additive model. The minor allele “G” of rs662799 was associated with a decreased ischemic stroke risk in the additive model. Additionally, strong linkage was found in 3 blocks constituted by rs1042034, rs676210, rs693, rs673548 in *APOB*; rs3791981, rs679899 in *APOB*; and rs651821, rs662799, rs17120035 in *APOA5*. Our data suggested that gene polymorphisms in the *APO* genes may exert influences ischemic stroke susceptibility in a Chinese Han population.

## INTRODUCTION

In recent years, ischemic stroke is becoming the most seriously disabling illness in industrialized countries [1]. As the population growth and aging in the world, the incidence of ischemic stroke is increasing every year. We have to take action now to look for some novel targets for new therapeutic strategies [2]. Previous study have suggested that the ischemic stroke is a multifactorial disease which is influenced by many factors, such as age, gender, obesity, smoking status, history of hypertension, diabetes and abnormal lipid metabolism as well as gene variation [3, 4]. Recent studies have identified several predisposing genes that are associated with ischemic stroke risk, including *HDAC9* [5], *LTC4S*, *ALOX5* [6], *APOA1*, *APOB* [7].

The protein encoded by *APOA1* is the major protein component of high density lipoprotein (HDL) in plasma. Defects in *APOA1* are associated with HDL deficiencies, including Tangier disease and systemic non-neuropathic amyloidosis [8]. *APOA1* is closely linked with *APOA5*, which is another apolipoprotein gene on chromosome 11. The apolipoprotein encoded by *APOA5* regulates the plasma triglyceride levels. Previous study have found that *APOA5* mutations are associated with hypertriglyceridemia and hyperlipoproteinemia type 5 [9].

The protein encoded by *APOB* is the main apolipoprotein of chylomicrons and low density lipoproteins (LDL). Mutations in *APOB* or its regulatory region cause hypobetalipoproteinemia, normotriglyceridemic hypobetalipoproteinemia and hypercholesterolemia [10]. Additionally, APO levels have a closely correlation with lipid levels in the human body, and APOB/APOA1 could be used for predicting of abnormal lipid metabolism related disease [7].

To date, several independent studies suggest that APOA1, APOA5 and APOB levels are risk factors for ischemic stroke risk in Western populations. However, little is known about the contribution of *APOA1*, *APOA5* and *APOB* genes polymorphisms to ischemic stroke risk, especially in the Chinese Han population. We therefore performed a case-control study to investigate the associations between single nucleotide polymorphisms (SNPs) in *APOA1*, *APOA5* and *APOB*, and the risk of ischemic stroke in Chinese Han population.

## RESULTS

This study included 488 ischemic stroke cases (325 men, 163 women; mean age 63.96 ± 11.06 years) and 503 healthy controls (196 men, 308 women; mean age 50.36 ± 7.89 years). The clinical characteristics of cases and controls are shown in Table 1. Age (*p* < 0.001) and sex (*p* < 0.001) were significantly different between cases and healthy controls. Multivariate analyses were adjusted for age and sex.

**Table 1:** Basic characteristics of case and control subjects

Variables	Case (*n*= 488)		Control (*n*=503)		*p* value
	No.	%	No.	%	
Sex					
Male	325	66.6	195	38.9	*p*<0.001a
Female	163	33.4	308	61.1	
Age					
Mean ± SD	63.96 ± 11.06		50.36 ± 7.89		*p* <0.001b

*^a^P* values were calculated by two-sided chi-square tests;

*^b^P* values were calculated from Student t tests.

To design the multiplexed SNPs MassEXTEND assay, we used Sequenom MassARRAY Assay Design 3.0 Software (PCR primers are shown in Table 2). The minor allele frequencies (MAFs) of the analyzed SNPs in the case and control groups are shown in Table 3. All SNPs were in Hardy-Weinberg equilibrium (HWE) in the controls (*p* > 0.05). We found three SNPs were significantly associated with ischemic stroke: rs693 with a *p* value of 0.042 (OR = 1.406; 95%CI = 1.011-1.956), rs651821 with a *p* value of 0.007 (OR = 0.760; 95%CI = 0.622-0.929) and rs662799 with a *p* value of 0.006 (OR = 0.755; 95%CI = 0.618-0.923).

**Table 2:** PCR primers used for this study

SNP_ID	1st-PCR primer sequences	2nd-PCR primer sequences	UEP sequences
rs1042034	ACGTTGGATGATGAAGATTAAGGCATAGG	ACGTTGGATGATCCAAGATGAGATCAACAC	ATGAGATCAACACAATCTTCA
rs1801702	ACGTTGGATGTCCTTTCGAGTTAAGGAAAC	ACGTTGGATGGGCTTTAAATACCTCTTGGG	TGATAAATCTTTCAACAGTTCC
rs676210	ACGTTGGATGATAGCTTGCCAAAAGTAGG	ACGTTGGATGTTTTCAAGTTCCTGACCTTC	ggtccAGTTCCTGACCTTCACATAC
rs693	ACGTTGGATGGGTATCGTTGAAGTTCCTGC	ACGTTGGATGCACATGAAGGCCAAATTCCG	aGCCAAATTCCGAGAGAC
rs673548	ACGTTGGATGCTTTCAGTGCATTGTCCAG	ACGTTGGATGAAGAGCAATGAACATTAGGC	GAACATTAGGCAAAAATACC
rs3791981	ACGTTGGATGCTACCTAGCTACCTCAAATC	ACGTTGGATGGTTTTGAGAATGAAGAAACA	AGAATGAAGAAACAATAGCTC
rs679899	ACGTTGGATGTCCATGACAGTTGGAAGTTG	ACGTTGGATGATAACATGGTGTGTCAGCTC	CTGAAAAAGTTAGTGAAAGAAG
rs512535	ACGTTGGATGTTCCGGTGGGAAATGGGCAG	ACGTTGGATGCCTCATAGACATCTGGAACC	aCATGCATCGTTTCCTTC
rs651821	ACGTTGGATGCTCCCTCCACCTGTCTTCT	ACGTTGGATGAGACCCACCTGAAAGAAGAG	CAGCCATGCTTGCCATTA
rs662799	ACGTTGGATGAGCATTTGGGCTTGCTCTCC	ACGTTGGATGTCTGAGCCCCAGGAACTGGA	cctGAACTGGAGCGAAAGT
rs17120035	ACGTTGGATGTACACACGTTCACAAGCTCC	ACGTTGGATGCTGGTGCAATGATGGTAGTG	GGATTGATTCAAGATGCATTTA
rs9804646	ACGTTGGATGCTGGGTTCTGATTCTGGTTG	ACGTTGGATGGTTTGAGGAGATCAAGTGGC	ACGTTGGATGCTTTCAGTGCATTGTCCAG
rs5072	ACGTTGGATGTGTGACCCTGCCTGGAGAT	ACGTTGGATGCCGAGTCCTCACCTAATATC	gaatATGGTCTGGATGGAGAAAC
rs632153	ACGTTGGATGAGCTGTGCTCCTGGAGGCTG	ACGTTGGATGAGGGACATGAGCAACCCTTC	AGCTGGAGAAGGCAAAG

**Table 3:** Allele frequencies in cases and controls and odds ratio estimates for ischemic stroke risk (adjusted for gender and age)

SNP ID	Chromosome	Position	Allele	Gene	Role	HWE *p*	MAF		OR(95%CI)	*p* value
			A/B				Case	Control		
rs1042034	2	21225281	T/C	*APOB*	Coding exon	0.1247	0.297	0.282	1.075(0.885-1.305)	0.467
rs1801702	2	21225485	G/C	*APOB*	Coding exon	1	0.027	0.029	0.922(0.539-1.577)	0.767
rs676210	2	21231524	G/A	*APOB*	Coding exon	0.1534	0.295	0.283	1.057(0.870-1.284)	0.577
rs693	2	21232195	A/G	*APOB*	Coding exon	0.2639	0.091	0.067	1.406(1.011-1.956)	0.042*
rs673548	2	21237544	G/A	*APOB*	Intron (boundary)	0.1526	0.295	0.284	1.056(0.869-1.283)	0.581
rs3791981	2	21245367	G/A	*APOB*	Intron	0.1267	0.046	0.051	0.905(0.600-1.365)	0.634
rs679899	2	21250914	G/A	*APOB*	Coding exon	0.8762	0.184	0.173	1.081(0.859-1.361)	0.505
rs512535	2	21267782	T/C	*APOB*	Promoter	0.7202	0.256	0.249	1.041(0.850-1.275)	0.696
rs651821	11	116662579	C/T	*APOA5*	5′ UTR	0.9139	0.238	0.291	0.760(0.622-0.929)	0.007*
rs662799	11	116663707	G/A	*APOA5*	Promoter	1	0.238	0.292	0.755(0.618-0.923)	0.006*
rs17120035	11	116663851	T/C	*APOA5*	Promoter	1	0.100	0.100	1.000(0.746-1.341)	0.999
rs9804646	11	116665079	T/C	*APOA5*	Promoter	0.8933	0.221	0.211	1.064(0.859-1.318)	0.567
rs5072	11	116707583	A/G	*APOA1*	Intron	0.5463	0.333	0.333	1.001(0.831-1.207)	0.988
rs632153	11	116710239	T/G	*APOA1*	Promoter	0.4523	0.065	0.065	1.001(0.700-1.433)	0.995

SNP single nucleotide polymorphism, HWE Hardy-Weinberg equilibrium, MAF minor allele frequency, OR odds ratio, CI confidence interval.

*p ≤ 0.05 indicates statistical significance.

We further assessed the association between each SNP and ischemic stroke risk in an unconditional logistic regression analysis, which was performed using three models: additive, dominant and recessive model (Table 4). The minor allele “A” of rs693 in *APOB* was associated with an increased ischemic stroke risk in the additive model (OR = 1.583; 95 % CI = 1.045 - 2.397; *p* = 0.030) and dominant model (OR = 1.610; 95 % CI = 1.024 - 2.530; *p* = 0.039) respectively. The minor allele “C” of rs651821 in *APOA5* was associated with a decreased ischemic stroke risk in the additive model (OR = 0.773; 95 % CI = 0.597 - 0.999; *p* = 0.040). The minor allele “G” of rs662799 in *APOA5* was associated with a decreased ischemic stroke risk in the additive model (OR = 0. 768; 95 % CI = 0.593 - 0.993; *p* = 0.044).

**Table 4:** Association between APO SNPs and ischemic stroke risk in multiple inheritance models (adjusted for gender and age)

SNP ID	Minor Allele	Additive model		Dominant model		Recessive model		
		OR (95% CI)	*p* value	OR (95% CI)	*p* value	OR (95% CI)	*p* value	
rs1042034	T	1.143 (0.895-1.461)	0.284	1.218 (0.881-1.684)	0.233	1.111 (0.642-1.923)		0.707
rs1801702	G	0.962 (0.483-1.917)	0.912	0.940 (0.463-1.909)	0.864	-		0.999
rs676210	G	1.119 (0.875-1.431)	0.372	1.196 (0.865-1.653)	0.280	1.046 (0.600-1.823)		0.874
rs693	A	1.583 (1.045-2.397)	0.030*	1.610 (1.024-2.530)	0.039*	2.685 (0.484-14.890)		0.258
rs673548	G	1.109 (0.867-1.419)	0.409	1.180 (0.853-1.632)	0.319	1.044 (0.599-1.818)		0.880
rs3791981	G	0.770 (0.457-1.297)	0.326	0.792 (0.450-1.393)	0.419	0.276 (0.022-3.536)		0.323
rs679899	G	1.019 (0.757-1.372)	0.901	1.059 (0.751-1.492)	0.745	0.795 (0.316-2.003)		0.627
rs512535	T	0.971 (0.748-1.262)	0.828	0.965 (0.751-1.492)	0.829	0.965 (0.502-1.857)		0.915
rs651821	C	0.773 (0.597-0.999)	0.040*	0.743 (0.536-1.028)	0.073	0.659 (0.352-1.231)		0.190
rs662799	G	0.768 (0.593-0.993)	0.044*	0.735 (0.531-1.017)	0.063	0.660 (0.353-1.233)		0.193
rs17120035	T	0.999 (0.688-1.453)	0.998	0.971 (0.644-1.462)	0.887	1.472 (0.323-6.588)		0.613
rs9804646	T	1.071 (0.812-1.413)	0.626	1.056 (0.758-1.472)	0.746	1.261 (0.589-2.700)		0.551
rs5072	A	0.915 (0.716-1.170)	0.480	0.869 (0.627-1.204)	0.398	0.961 (0.567-1.627)		0.882
rs632153	T	1.028 (0.641-1.651)	0.908	1.050 (0.644-1.711)	0.844	0.355 (0.008-16.740)		0.599

SNP single nucleotide polymorphism, OR odds ratio, CI confidence interval.

*p ≤ 0.05 indicates statistical significance.

**Figure 1:**
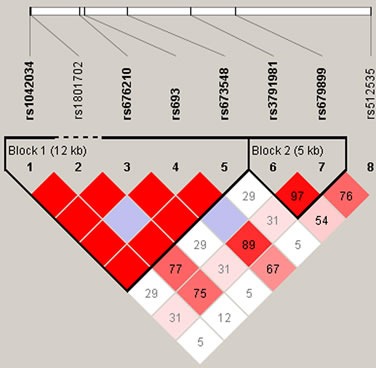
D' linkage map for the 8 SNPs in *APOB*

**Figure 2:**
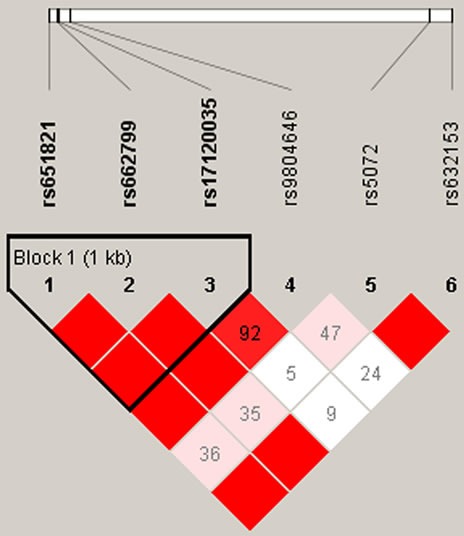
D' linkage map for the 6 SNPs in APOA1 and APOA5 A standard color scheme was used to display linkage disequilibrium (LD), with bright red corresponding to strong LD (LOD = 2, D' = 1), white corresponding to no LD (LOD < 2, D' < 1), and pink/red (LOD = 2, D' < 1) and blue (LOD < 2, D' = 1) corresponding to intermediate LD.

We further characterized the SNPs in *APOs* using linkage disequilibrium (LD) and haplotype analyses. Figure 1 showed the two blocks in *APOB* constructed by rs1042034, rs676210, rs693, rs673548 and rs3791981, rs679899 in chromosome 2 with D' = 1. Figure 2 showed the block in *APOA5* constructed by rs651821, rs662799 and rs17120035 in chromosome 11 with D' = 1. The association analysis between the three blocks and ischemic stroke risk was shown in Table 5. The haplotype “TGAG” in *APOB* constructed by rs1042034, rs676210, rs693 and rs673548 in chromosome 2 was associated with an increased ischemic stroke risk (OR = 1.583; 95 % CI = 1.045 - 2.397; *p* = 0.031). The haplotype “CGC” in *APOA5* constructed by rs651821, rs662799 and rs17120035 in chromosome 11 was associated with a decreased ischemic stroke risk (OR = 0.770; 95 % CI = 0.595 - 0.997; *p* = 0.047).

**Table 5:** Haplotype frequencies of APO genes and the association with ischemic stroke risk in cases and control (adjusted for gender and age)

Chromosome	Gene	SNPs	Haplotype	Frequency	Frequency	OR	95% CI		*p* value
				Case	Control				
chr2	*APOB*	rs1042034|rs676210|rs693|rs673548	TGAG	0.091	0.067	1.583	1.045	2.397	0.030*
			TGGG	0.203	0.216	0.940	0.714	1.238	0.659
			CAGA	0.701	0.717	0.873	0.683	1.115	0.276
chr2	*APOB*	rs3791981|rs679899	GG	0.046	0.050	0.774	0.459	1.306	0.338
			AG	0.138	0.123	1.139	0.815	1.592	0.447
			AA	0.816	0.826	0.984	0.731	1.324	0.913
chr11	*APOA5*	rs651821|rs662799|rs17120035	TAT	0.100	0.100	1.000	0.688	1.453	0.998
			CGC	0.238	0.291	0.770	0.595	0.997	0.047*
			TAC	0.662	0.607	1.245	0.985	1.573	0.067

SNP single nucleotide polymorphism, OR odds ratio, CI confidence interval.

*p ≤ 0.05 indicates statistical significance.

## DISCUSSION

In the current study, we evaluated the association between fourteen SNPs in three *APO* genes and ischemic stroke risk in the Chinese Han population. We found the minor allele “A” of rs693 was associated with an increased ischemic stroke risk. The minor allele “C” of rs651821 and “G” of rs662799 have a protective role for ischemic stroke.

The SNP rs693 at chromosome 2p24.1 was located in the exon region of *APOB* gene. The minor allele “A” of rs693 in codon 3611 resulted in amino acid substitution of glutamine to arginine, which could further changed the LDL-receptor binding affinity [11]. Previous studies demonstrated rs693 have a closely correlation with cholesterol and LDL-cholesterol levels [12–15]. Our study demonstrated rs693 was associated with ischemic stroke risk in a Chinese Han population, the detailed mechanism of how the SNP affected the progressing of ischemic stroke deserved further investigation.

The SNP rs651821 and rs662799 at chromosome 11q23.3 was located in the 5′ UTR and promoter region of *APOA5* gene respectively. As demonstrated by previous studies, the elevated triglyceride level may be an independent risk factor for ischemic stroke [16]. Human and animal data consistently show that the newly identified *APOA5* gene may play an important role in the development of ischemic stroke and triglyceride metabolism [17, 18]. APOA5-1131T/C (rs662799), as one polymorphism site of *APOA5*, has been widely studied in ischemic stroke susceptibility. However, inconclusive results have been obtained. Some studies supported the conclusion that risk for ischemic stroke was associated with the polymorphism, whereas other studies drew converse conclusions [17–22]. Our study supported rs662799 was associated with ischemic stroke risk in a Chinese Han population, further meta-analysis will be need to confirm the results.

The haplotype analysis suggested that the combination of certain SNPs could increase or decrease the risk of ischemic stroke since the change of a certain SNP may affect the change of another SNP thus generating a joint effect in the progressing of ischemic stroke. The detailed mechanism of this phenomenon deserved further investigation.

Our study had several intrinsic limitations. For example, ischemic stroke is a very complicated process, and environment factors such as eating and exercise habits are important risk factors for ischemic stroke. Because our study had a relatively small sample size, and it did not incorporate data regarding eating and exercise habits, we could not explore the interactions between genetic polymorphisms and environmental factors in ischemic stroke. Therefore, the relationship between *APOs* polymorphisms and environment factors in ischemic stroke must be evaluated in future studies.

In sum, our present study provided evidence that three SNPs rs693, rs651821 and rs662799 were associated with ischemic stroke risk in a Chinese Han population, which could be used in clinical diagnosis of ischemic stroke. Further investigations are deserved to discover more susceptible loci to other cardiovascular diseases, and to clarify the molecular mechanism of ischemic stroke.

## MATERIALS AND METHODS

### Study subjects

All the participants in this study were Han Chinese lived in Hainan Province. A total of 488 patients who diagnosed with ischemic stroke were recruited from the Haikou People's Hospital between January 2013 and February 2016. They were all newly diagnosed to be ischemic stroke patients according to the International Classification of Disease (9th revision, codes 430 to 438) on the basis of history, clinical symptoms, physical examination, and cranial computed tomography or magnetic resonance imaging. Patients with hemorrhagic stroke, subarachnoid hemorrhage, transient ischemic attack, traumatic brain injuries, infectious diseases, and tumors were excluded in this study.

We also recruited 503 controls at the Haikou People's hospital. Controls were healthy, unrelated individuals selected randomly from the medical examination center of the hospital. All participants were informed of the procedures and purpose of the study, and each participant provided signed informed consent forms. The use of human samples in this study was approved by the Ethics Committees of the local participating hospitals and was in accordance with Department of Health and Human Services (DHHS) regulations for protection of human research subjects.

### Polymorphisms selection and genotyping assays

Candidate SNPs in the *APO* genes were selected from previously published polymorphisms associated with stroke [23, 24]. Validated tSNPs were selected with a MAF > 5% in the HapMap CHB population. A total of 14 SNPs in the *APO* genes including 2 SNPs in *APOA1*, 4 SNPs in *APOA5* and 8 SNPs in *APOB* were selected for further genotyping. Genomic DNA was extracted from peripheral blood leukocytes using the GoldMag® nanoparticles method (GoldMag Ltd. Xi'an, China) according to the manufacturer's instructions and DNA concentration was measured using the NanoDrop 2000 (Thermo Scientific, Waltham, Massachusetts, USA). We used Sequenom MassARRAY Assay Design 3.0 Software to design Multiplexed SNP MassEXTEND assays [25]. SNP genotyping was performed using the Sequenom MassARRAY RS1000 with a standard protocol recommended by the manufacturer [25]. Data management and analyses were performed using the Sequenom Typer 4.0 software as previously described [25, 26].

### Statistical analysis

Statistical analyses were performed using Microsoft Excel (Redmond, WA, USA) and SPSS 16.0 statistical package (SPSS, Chicago, IL, USA). All *p* values in this study were two-sided, and *p* ≤ 0.05 was considered as the statistical significance threshold [27]. An exact test was used to assess the departure of each SNP frequency from Hardy-Weinberg equilibrium (HWE) in controls. We compared allele frequencies between cases and controls using a χ2 test. *p* values were calculated using unconditional logistic regression analysis with adjustment for age and gender [28, 29].

The three genetic models (dominant, recessive and additive) were applied by PLINK software (http://www.cog-genomics.org/plink2/) to assess the association of single SNPs with the risk of ischemic stroke. The odds are a way of representing probability, especially familiar for betting. The confidence intervals are standard errors for the log odds ratio. The odds ratios (ORs) and 95% confidence intervals (95% CIs) were calculated by unconditional logistic regression analyses adjusted for age and sex [30].

We used the Haploview software package (version 4.2) to analyze the linkage disequilibrium (LD), haplotype construction, genetic association at polymorphism loci and a D' value greater than 0.8 indicated that the related SNPs formed one block [31]. Marker information, including name and location is loaded separately. The program filters out markers which fall below 5% for subsequent steps. Haplotypes with frequencies > 1% were selected for analysis of ischemic stroke risk.
